# Extracts of endophytic fungus xkc-s03 from *Prunella vulgaris* L. spica inhibit gastric cancer *in vitro* and *in vivo*

**DOI:** 10.3892/ol.2014.2722

**Published:** 2014-11-20

**Authors:** JIANAN TAN, HAIZHI QI, JIANGDON NI

**Affiliations:** Department of Surgery, Second Hospital of Zhongnan University, Changsha, Hunan 410011, P.R. China

**Keywords:** gastric cancer, *Prunella vulgaris* L., endophytic fungi, ethyl acetate extract

## Abstract

*Prunella vulgaris* L. belongs to the *Prunella* genus and has been proven effective in the treatment of gastric cancer, however, the therapeutic activity of the endophytic fungi is not yet well understood. The results of the present study suggest that the ethyl acetate extract (S03-EA) of the endophytic fungus XKC-S03, isolated from *Prunella vulgaris* L. spica, is a potent anticancer agent with the potential to treat gastric cancer. In the present study, the effects of S03-EA on gastric cancer *in vitro* and *in vivo* were determined using the 1-(4,5-dimethylthiazol-2-yl)-3,5-diphenylformazan assay and the human gastric cancer SGC 7901 cell xenograft model. The tumor tissue was fixed with 10% formaldehyde solution and the levels of the apoptotic proteins, B-cell lymphoma protein-2 (Bcl-2), Bcl-2-associated X protein (Bax) and pro-angiogenic vascular endothelial growth factor (VEGF), were measured by immunohistochemistry. The results indicated that treating SGC 7901 cells with petroleum ether (S03-PE), ethyl acetate (S03-EA) or dichloromethane (S03-DM) extracts from the XKC-S03 fermentation broth inhibited cell proliferation. S03-EA demonstrated the best activity, with a half maximal inhibitory concentration of 25.89 μg/ml and dose-dependent suppression of the SGC 7901 tumor cells *in vivo,* without any evident adverse effects. In addition, the 100-mg/kg/day S03-EA-treated tumor tissue revealed a downregulation of Bcl-2 and VEGF expression and an upregulation of Bax expression. In conclusion, the S03-EA extract of XKC-S03, isolated from *Prunella vulgaris* L. spica, exhibits a growth-suppressive activity on gastric cancer *in vitro* and *in vivo*.

## Introduction

The increase in the incidence of cancer has become a worldwide health concern, with gastric cancer accounting for a large proportion of cancer-related mortalities. A global annual case-fatality ratio (CFR) of 0.75 exists for gastric cancer, with ~934,000 new cases diagnosed and 700,349 associated fatalities. The CFR of gastric cancer exceeds that of colon (CFR, 0.52), breast (CFR, 0.36) and prostate (CFR, 0.33) cancer (1,2). In 2006, gastric cancer was the fifth most common newly diagnosed cancer and the fourth most common cause of mortality in Europe (3). The majority of gastric cancer patients are diagnosed with locally advanced or metastatic disease. Furthermore, gastric cancer commonly has a poor prognosis, particularly if it localized to the cardia and gastroesophageal junction. It is estimated that 40–60% of patients undergoing treatment for tumor resection, develop recurrence (4). Despite this, using chemotherapy to treat other non-hematological cancers, such as breast and colorectal cancer, has taken priority over gastric cancer in recent decades. Therefore, a requirement exists for renewable, environmentally friendly and accessible therapeutic agents to treat gastric cancer (5).

*Prunella vulgaris* L. belongs to the *Prunella* genus (Lamiaceae) and has been used worldwide for centuries as an alternative medicine in the treatment of fungal and bacterial infections. In addition, anti-oxidant (6,7), anti-allergy (8), immunomodulatory (9), anti-diabetic (10) and anticancer (including lymphoma (11), breast cancer (12) and esophageal cancer) (13) activities have been observed in recent years. As previously reported, the injection of *Prunella vulgaris* L. induces a significant effect on the human gastric cancer SGC 7901 cell line (14), however, few studies have been performed on the endophytic fungi XKC-S03, extracted from the *Prunella vulgaris* L. plant. In addition, compared with their counterparts derived from conventional medicinal plants, the use of active metabolites from endophytic fungi has a number of advantages. These include minimal destruction of resources, sustainable utilization, straightforward large-scale industrial production and simple quality control. At present, an increasing number of compounds with various bioactivities are being isolated from endophytic fungi (15–18). Therefore, the objective of the present study was to investigate the therapeutic potential of the endophytic fungi from *Prunella vulgaris* L. *in vivo* and *in vitro*, and to examine the molecular targets for treating gastric cancer.

## Materials and methods

### Samples

During summer 2009, the endophytic fungus XKC-S03 was isolated from the spicae of *Prunella vulgaris* L., which were collected from the Second Hospital of Zhongnan University, Changsha, China. In total, 10 liters of XKC-S03 fermentation broth was isolated using 30 liters of petroleum ether (S03-PE), ethyl acetate (S03-EA), dichloromethane (S03-DM) and n-butyl alcohol (S03-BA) (all obtained from Santa Cruz Biotechnology, Inc., Santa Cruz, CA, USA), to obtain 3.9-, 10.3-, 3.0- and 7.2-g extracts, respectively.

### Reagents

The 3-(4,5-dimethylthiazol-2-yl)-2,5-diphenyltetrazolium bromide (MTT) assay was purchased from Amresco (Solon, OH, USA). Polyclonal rabbit anti-human vascular endothelial growth factor (VEGF; dilution 1:2,000; catalogue number, ab9571), polyclonal mouse anti-human B-cell lymphoma protein-2 (Bcl-2; dilution, 1:3,000; catalogue number, ab117115) and polyclonal rabbit anti-human Bcl-2-associated X protein (Bax; dilution, 1:2,000; catalogue number, ab7977) antibodies were purchased from Abcam (Cambridge, UK). Cyclophosphamide (CTX) was purchased from the National Institutes for Food and Drug Control, (Beijing, China).

### Cell lines and culture

The SGC-7901 cell line was purchased from the Beijing Institute for Cancer Research (Beijing, China) and maintained in RMPI 1640 medium (Beijing Solarbio Science and Technology Co., Ltd., Beijing, China), which contained 100 U/ml penicillin and 100 mg/ml streptomycin. The medium was supplemented with 15% fetal bovine serum (Zhejiang Tianhang Biological Technology Co., Ltd., Hangzhou, China) and cultures were incubated at 37°C in a humidified atmosphere of 5% CO_2_.

### Cell proliferation assay

Cell proliferation was analyzed using the MTT assay as previously described (19). After 12h, the SGC 7901 cells (1×10^5^) were seeded into a 96-well plate and equal volumes of different concentrations of XKC-S03 extracts (25, 50, 100, 250 and 500 μg/ml) were added to the cells for 24, 48 and 72 h. Following this, MTT (final concentration, 5 mg/ml) was added and incubated at 37°C for 4 h. The medium was removed, formazan was dissolved in dimethylsulfoxide and the optical density was measured at 570 nm using an ELISA plate reader (MQX200; BioTek Co., Ltd, Winooski, CA, USA). All the experiments were performed in triplicate, and three independent experiments were conducted. The rate of cell viability to control (I%) was calculated as: I% = A570(treated)/A570(control) × 100 (20). CTX and phosphate-buffered saline were used as positive and negative controls, respectively.

### Antitumor activity of S03-EA in vivo

In total, 40 male nude BALB/c mice, aged 6–8 weeks old and weighing between 20 and 22 g, were obtained from the Animal Research Center, Capital Medical University (Beijing, China). The SGC-7901 cells (5×10^6^ in 0.5 ml 0.9% saline) were subcutaneously injected into the flanks of the mice. When the tumors reached 0.2–0.3 cm^3^, the mice were randomly assigned to one of three groups. For the treatment group, 50 or 100 mg/kg/day S03-EA was administered daily for 15 days by intraperitoneal (i.p.) injection. The negative and positive control groups were treated with 0.9% saline and CTX, respectively, at a dose of 10 mg/kg/day every 3 days, according to the same schedule. On the last day, the mice in the treatment and control groups (n=10 in each group) were anaesthetized with pentobarbital sodium intravenously, and sacrificed. The tumor volumes were recorded using Vernier calipers every two days and calculated by the following equation: V = ab^2^/2, where ‘a’ represents the length and ‘b’ represents the width (21), and then transformed into relative values (V) using the formula: V = V_t_/V_0_, where V_0_ is the initial tumor volume and Vt is the final tumor volume after sacrifice (22). The body and tumor weight of the mice were recorded following 15 days of treatment. All surgical procedures and care administered to the animals were in accordance with institutional guidelines of the of Second Hospital of Zhongnan University. The study was approved by the Committee on the Use of Live Animals in Teaching and Research of Capital Medical University (Beijing, China).

### Observation of Bcl-2, Bax and VEGF expression in tumor cells treated with S03-EA

Two slices of the tumor tissue were selected randomly from each treatment group and the cells were observed in five fields. The expression rates of cells positive for Bcl-2, Bax and VEGF were calculated to obtain a conclusion. Paraffin sections were performed as previously described (23) and immunostained by the streptavidin-peroxidase conjugate method.

### Statistical analysis

The results are expressed as the mean ± standard deviation and are representative of at least three independent experiments. The individual comparisons were obtained by Duncan’s multiple range test subsequent to the demonstration of homogeneity of variance with a one-way analysis of variance for more than two groups using SPSS version 13.0 (SPSS, Inc., Chicago, IL, USA). P<0.001 was considered to indicate a statistically significant difference between groups.

## Results

### S03-EA inhibits the proliferation of SGC 7901 cells in vitro

**Figure 1:**
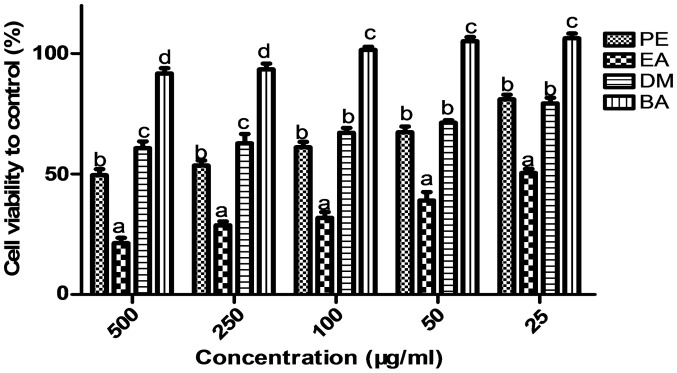
Four different extracts of XKC-S03 inhibit the proliferation of the SGC 7901 cell line. SGC 7901 cells were exposed to serial dilutions of S03-PE, -EA, -DM and -BA (25–500 μg/ml) for 24, 48 or 72 h followed by analysis by MTT assay. Values not sharing a common superscript (^a^, ^b^, ^c^ and ^d^) differ significantly (Duncan’s multiple range test). Results are expressed as the mean ± standard deviation (n=3), P<0.001 denotes statistically significant differences. PE, petroleum ether; EA, ethyl acetate; DM, dichloride methane; BA, n-butyl alcohol.

The extracts of XKC-S03 inhibited the growth of the human gastric cancer cells. The growth inhibitory effects of four different extractions of XKC-S03 (S03-PE, EA, DM and BA) on the human gastric cancer SGC 7901 cell line were evaluated using MTT assays. The SGC-7901 cells were treated with four crude extractions of XKC-S03 at concentrations of 500, 250, 100, 50 and 25 μg/ml. The results demonstrated that treatment with PE, EA and DM extractions induced growth inhibition in the SGC-7901 cells, and that S03-EA had the greatest effect on cell viability (Fig. 1), with a half maximal inhibitory concentration (IC_50_) of 25.89 μg/ml. The dose-dependent cell proliferation column diagram indicates that S03-EA is an effective growth inhibitor of the gastric cancer SGC 7901 cell line *in vitro* (Fig. 1).

### S03-EA inhibits the proliferation of SGC 7901 cells in vivo

**Figure 2:**
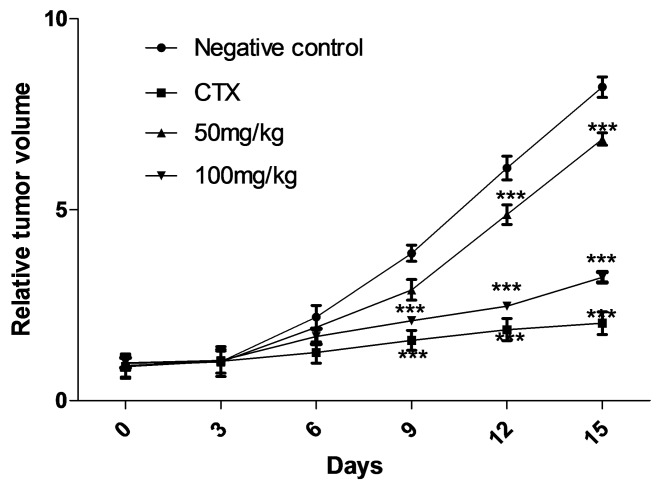
Relative SGC-7901-derived tumor volumes of the nude mice treated with 50 or 100 mg/kg S03-EA or with the positive (CTX) or negative (saline) controls. Tumor volumes were measured and transformed to relative tumor volume as detailed in the Materials and methods section. Relative tumor volume is shown as the mean ± standard deviation. ^***^P<0.001, denotes a significant difference compared with the negative control.

S03-EA showed dose-dependent suppression of SGC 7901 tumor growth compared with the negative saline control. At the end of the study (day 15), the established SGC 7901 xenograft mouse model (average tumor volume, 200 mm^3^) injected with i.p. S03-EA (50 or 100 mg/kg/day) demonstrated inhibition of tumor volume growth by 17.91 and 65.67%, and of tumor weight growth by 25.70 and 64.79%, respectively (P<0.001 vs. 0.9% saline controls). By day 15, the positive control, CTX, had reduced tumor volume and weight by 73.63 and 72.18%, respectively. No significant difference was identified between this result and that obtained with 100 mg/kg S03-EA (Table. I). Suppression of tumor growth was evident from the sixth day following treatment with S03-EA (Fig. 2), similar to the results observed in the CTX-treated group. In addition, no significant loss in body weight was identified in the S03-EA- and CTX-treated groups (Table I). The present *in vivo* study indicates that S03-EA is a potential therapeutic treatment of gastric cancer and is relatively non-toxic to nude mice.

### S03-EA downregulates Bcl-2 and VEGF expression, and actives Bax expression in gastric cancer cells

**Figure 3:**
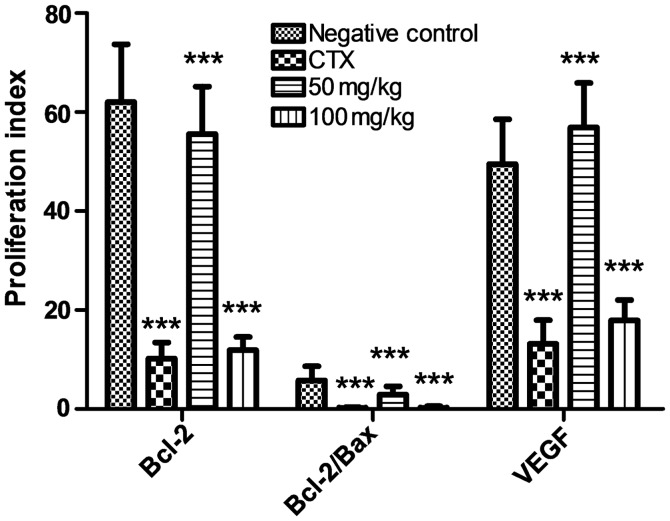
Proliferation index of the Bcl-2, Bax and VEGF proteins. Representative images of sections from each treatment group were stained with Bcl-2, Bax and VEGF antibodies, and counted to assess the proliferation. ^***^P<0.001, denotes a significant difference compared with the negative control. Bcl-2, B-cell lymphoma protein-2; Bax, Bcl-2-associated X protein; VEGF, vascular endothelial growth factor.

The effect of S03-EA on the expression levels of Bcl-2, VEGF and Bax was investigated to examine the mechanism behind its inhibition of gastric cancer cell proliferation. As shown in Fig. 3, Bcl-2 and VEGF expression in the 100-mg S03-EA- and CTX-treated groups were suppressed in the gastric cancer cells. However, the expression of Bax was higher in these treatment groups, which correlates with the decrease in the Bcl-2/Bax ratio observed. This result of the present study is consistent with previous studies investigating other tumors (24).

## Discussion

*Prunella vulgaris* L. has been demonstrated to possess a variety of anticancer activities. In addition, there have been studies investigating its endophytic fungi as a potential source of novel drug development. *Prunella vulgaris* L. was reported to be used in the treatment of gastric cancer as early as 1979 and revealed good curative effects (25). Subsequently, a *Prunella vulgaris* L. injection was developed by Shanghai Shuguang Hospital (Shanghai, China) and proved to be effective in the treatment of advanced gastric cancer and in SGC 7901 cells (14,26). In view of these studies, the endophytic fungi from *Prunella vulgaris* L was investigated in the present study. Firstly, the activity of the endophytes isolated from the spicae of *Prunella vulgaris* L. against the SGC 7901 cell line was screened. The crude extract of the XKC-S03 fermentation broth demonstrated the most promising result. In the present study, it was identified that the S03-EA extract of XKC-S03 inhibited SGC 7901 cell proliferation *in vitro* and tumor growth *in vivo*.

The results of the present study indicated that S03-EA suppressed SGC 7901 cell growth in a dose- and time-dependent manner, with an IC_50_ value of 25.89 μg/ml. A significant difference in inhibition activity was identified compared with the other three groups (S03-PE, -DM and -BA). According to the subsequent *in vivo* study, it was identified that the tumor volume and weight could be significantly reduced in mice following the sixth day of treatment with S03-EA. In addition, the average change in body weight subsequent to S03-EA treatment was not significant compared with that following CTX treatment (Table I). No gastrointestinal bleeding or other severe organ damage was identified in the S03-EA-treated mice, suggesting that it is safe for longer-term use. In order to explore the therapeutic mechanism of S03-EA-mediated inhibition, the expression of the three proteins, Bcl-2, Bax and VEGF, was analyzed in SGC 7901 cells.

The occurrence of tumors has a close association with apoptosis, a cellular event controlled by a balance between pro- and anti-apoptotic molecules, such as those belonging to the proto-oncogene Bcl-2 family. Among the Bcl-2 family, the Bcl-2 protein and its associated protein X (Bax) are delegates. In contrast to Bcl-2, Bax protein plays a pro-apoptotic role, activating apoptosis and initiating cell death (27,28). The results of the present study demonstrated that S03-EA inhibited the proliferation of the SGC 7901 cells by upregulating Bax expression and downregulating Bcl-2 expression. In addition, pro-angiogenic factors, such as VEGF, are also important to tumor cell proliferation, as they stimulate tumor progression, invasion and metastasis (29,30). In the present study, the suppression of VEGF expression may therefore have reduced the proliferation of the gastric cancer cells.

In summary, the present study demonstrated that treatment with the S03-EA extract of XKC-S03 isolated from *Prunella vulgaris* L. can suppress gastric cancer in mice. This finding lends support to existing studies on the therapeutic potential of active metabolites from *Prunella vulgaris* L., and the investigation of novel medicines for the treatment of gastric cancer.

## Figures and Tables

**Table I:** Tumor volume and weight of BALB/c nude mice treated with S03-EA and CTX.

Group	Body weight, g		Final tumor volume, cm^3^	Final tumor weight, g	Inhibition rate, % (volume/weight)

	Pre-treatment	Post-treatment			
Negative control	21.85±0.99	20.67±1.29	2.01±0.52	2.84±0.39	
CTX	21.46±0.63	31.49±2.26	0.53±0.12	0.9±0.28	73.63/72.18^a^
50 mg/kg	21.37±1.11	30.75±2.39	1.65±0.43	2.11±0.61	17.91/25.70^b^
100 mg/kg	20.11±0.68	28.68±3.12	0.69±0.21	1.00±0.38	65.67/64.79^a^

a P<0.0001;

b P<0.001.

S03-EA, ethyl acetate extract; CTX, cyclophosphamide.
